# Supplementary material for: Cell type-specific mapping of ion distribution in Arabidopsis thaliana roots
Source: Nat Commun. 2023 Jun 13;14:3351. doi: 10.1038/s41467-023-38880-0 (PMC10264424; doi:10.1038/s41467-023-38880-0)
Supplement: Supplementary file 1 — Supplementary Information [file 41467_2023_38880_MOESM1_ESM.pdf]

Supplementary information for

Cell type-specific mapping of ion distribution in *Arabidopsis thaliana* roots

Ricardo F. H. Giehl[1,*], Paulina Flis[2], Jörg Fuchs[1], Yiqun Gao[2], David E. Salt[2], Nicolaus von Wirén[1,*]

Author affiliations:

[1] Leibniz-Institute of Plant Genetics and Crop Plant Research (IPK) OT Gatersleben, 06466 Seeland, Germany.

[2] Future Food Beacon of Excellence & School of Biosciences, University of Nottingham, Nottingham LE12 5RD, UK.

* To whom correspondence may be addressed. Email: giehl@ipk-gatersleben.de or vonwiren@ipk-gatersleben.de.

a

Reagent	Solution A*	Mod. solution
KCl	40 mM	-
$MgCl_2$	8 mM	-
$CaCl_2$	8 mM	-
BSA	0.1%	-
MES	2 mM	2.5 mM
Mannitol	10.9%	5%

* Standard mixture according to Birnbaum et al. (2005)

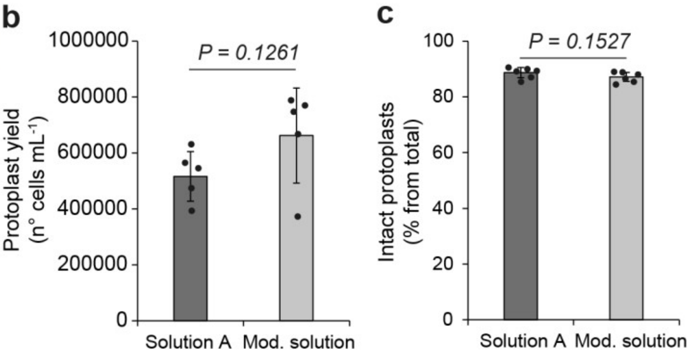

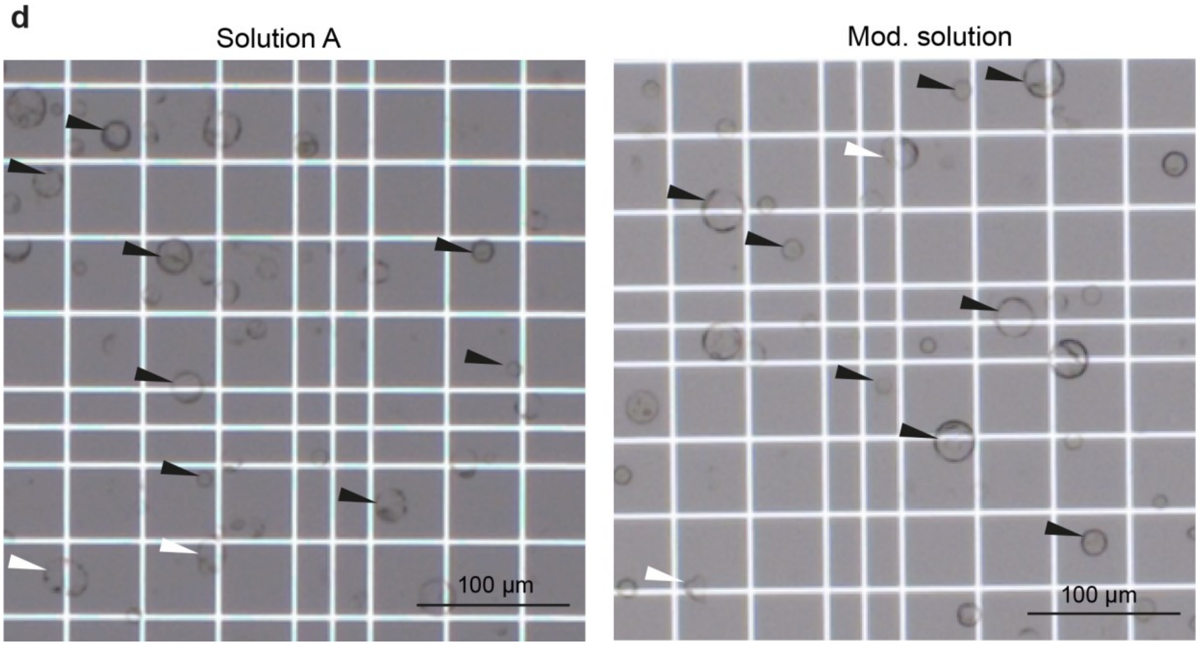

Supplementary Figure 1. Comparison of properties between standard and modified protoplasting solutions. a Elemental composition of standard solution A used to isolate protoplasts from *A. thaliana* roots and the solution used in this study (mod. solution). **b,c** Effect of standard solution A and modified solution on protoplast yield (**b**) and integrity (**c**). Data are means ± SD (n = 5 independent measurements in **b** or 6 independent measurements in **c**). **d** Appearance of protoplasts isolated with standard solution A or modified solution. Examples of burst or partially collapsed protoplasts are marked with white arrowheads while intact and perfectly spherical protoplasts are marked with black arrowheads. Shown are representative images of several independent isolations. Images are representative of experiments repeated at least two times. In **b**,**c**, *P*-values were determined by two-tailed unpaired Student's *t* test. Source data are provided as a Source Data file.

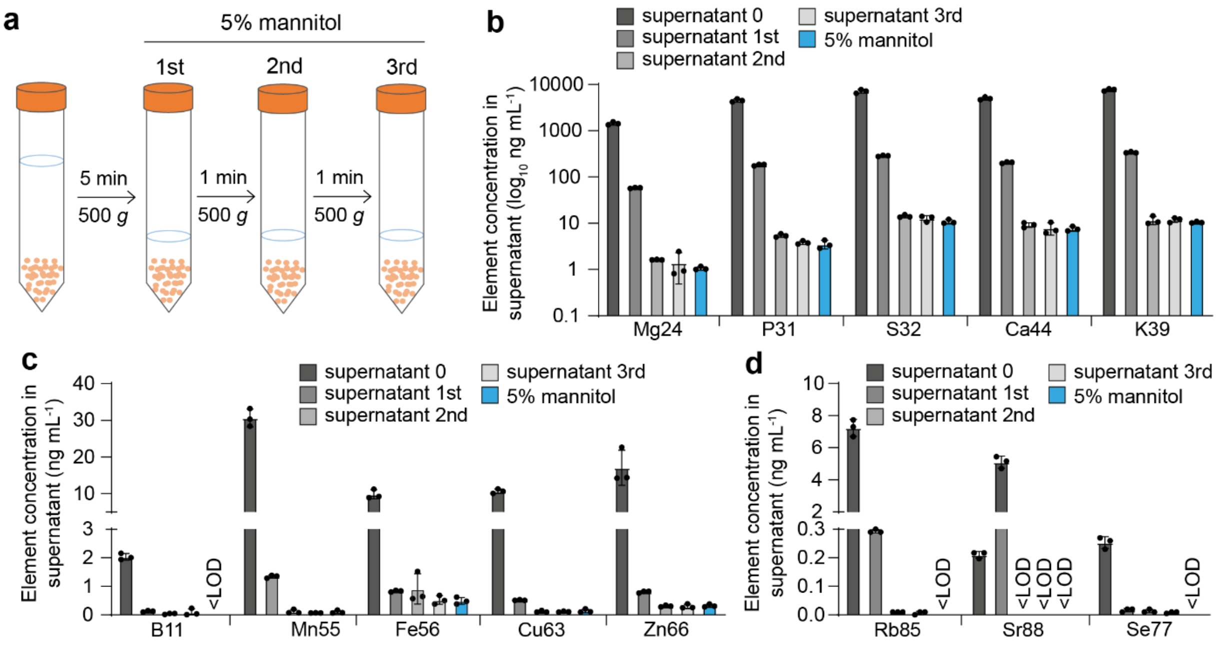

Supplementary Figure 2. Impact of protoplast washing on element concentrations. a Schematic representation of the procedure. After 90 min incubation in modified protoplast solution, samples were passed through a 40-µm cell strainer and the flow-through centrifuged at 500*g* for 5 min. The supernatant was discarded and protoplasts resuspended in ice-cold 5% mannitol (1st wash), and centrifuged at 500*g* for 1 min. This washing step was repeated up to three times. Samples of the supernatant were taken for ICP-MS analysis throughout the procedure. **b–d** To assess contamination contributed by the enzymes (powder), concentrations of the indicated macronutrients (**b**), micronutrients (**c**) and tracer elements (**d**) were assessed in the supernatant right after protoplast isolation (supernatant 0) or after subsequent washes of protoplast pellets with 5% mannitol resuspension buffer (supernatant 1st, 2nd and 3rd). Contamination brought in by the enzymes (powder) could be largely decreased after two consecutive washing steps. Bars represent means ± SD (*n* = 3 independent biological replicates). Note that, for macronutrients, concentrations are represented in log_{10} scale. < LOD = indicates that the concentration was below the limit of detection of the instrument. Source data are provided as a Source Data file.

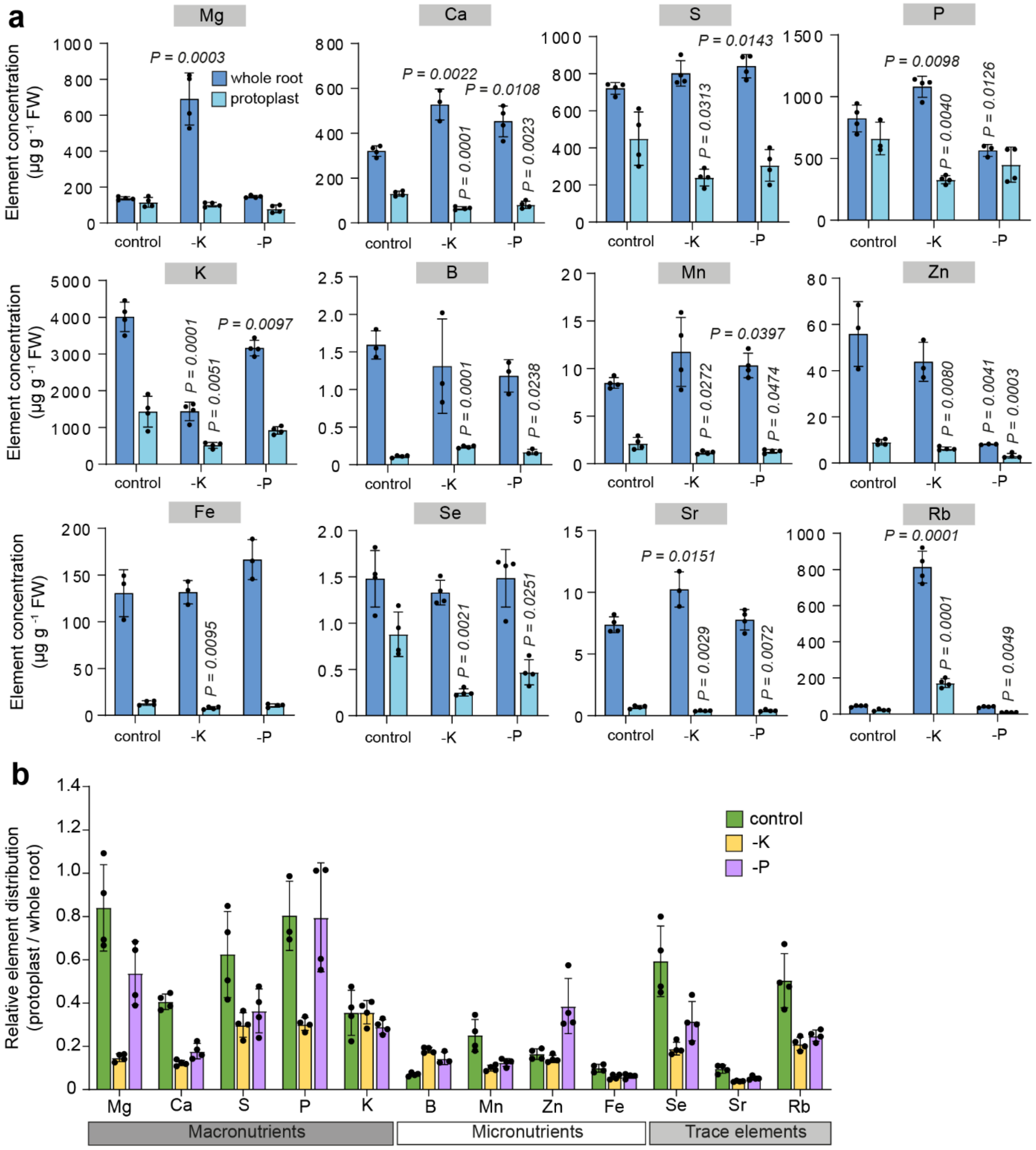

Supplementary Figure 3. Comparison of element concentrations in intact roots and in protoplasts isolated from *A. thaliana* roots. a Wild-type plants (Col-0) were grown for 5 days on solid agar containing sufficient levels of all nutrients (control) or deficient in K or P. The indicated elements were analyzed in whole roots or in protoplasts isolated from roots. K and Rb are also shown in Fig. 1. Bars represent means ± SD (n = 4 biological replicates, except for Ca concentration in whole roots of under -K, B concentration in whole roots of -K and -P, B concentration in protoplasts of -P, Zn and concentrations in whole roots of control, -K and -P, and Sr concentration in whole roots of -K, where n = 3 biological replicates). P-values were determined by two-tailed unpaired Student's t test (control *vs* -K or -P). FW, fresh weight. **b** Ratio of element levels in protoplasts to whole roots calculated from values shown in (*A*). Bars represent means ± SD (n = 4 biological replicates, except for P at -K and B at -P, where n = 3 biological replicates). Source data are provided as a Source Data file.

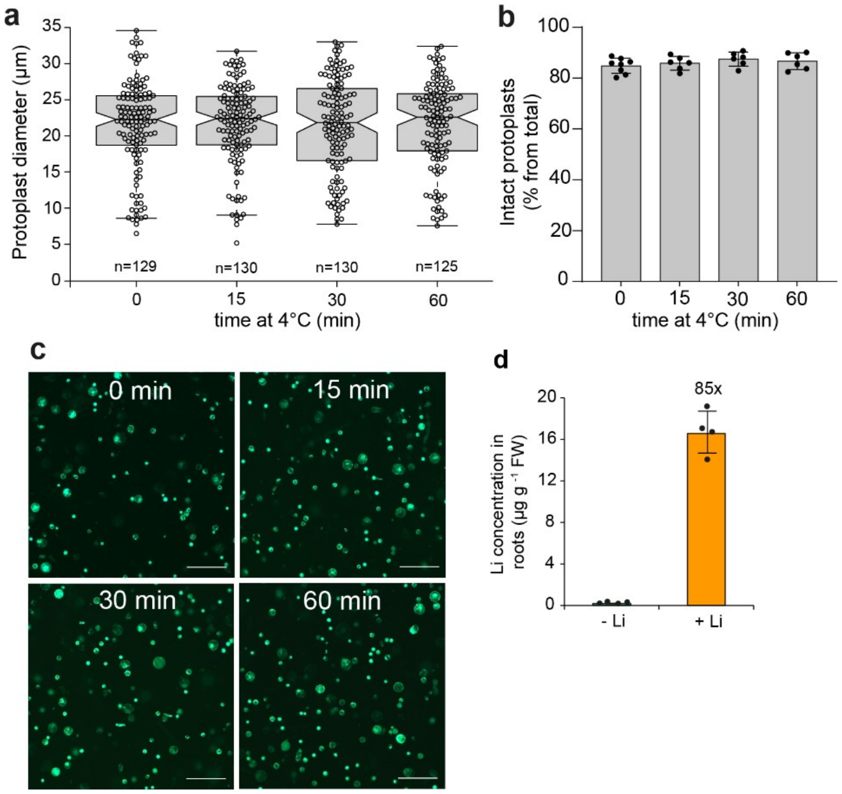

Supplementary Figure 4. Protoplasts isolated from roots of *pWER::GFP* reporter line using modified protoplast isolation solution. a–c Diameter of isolated protoplasts (combined GFP-expressing and non-expressing) (**a**), protoplast stability (**b**) and GFP signals (**c**) detected during time-course incubation in resuspension solution (5% mannitol, without enzymes and MES buffer) at 4°C. In the boxplots shown in **a**, boxes extend from the first to the third quartile around the median, while the ends of the whiskers indicate the maximum and minimum values within 1.5 x the interquartile range from the box ends. The number (*n*) of independent protoplasts analyzed is indicated. In **b**, bars represent means ± SD (*n* = 8, 6, 6 and 6 measurements from independent biological samples, respectively). Images are representative of experiments repeated at least two times. Scale bars, 100 µm. **d**, Li concentration in whole roots of five-day-old seedlings grown on solid half-strength MS agar media supplemented with 5 mM LiCl (+Li) or without added LiCl (-Li). Bars show means ± SD (*n* = 4 biological replicates). FW, fresh weight. Source data are provided as a Source Data file.

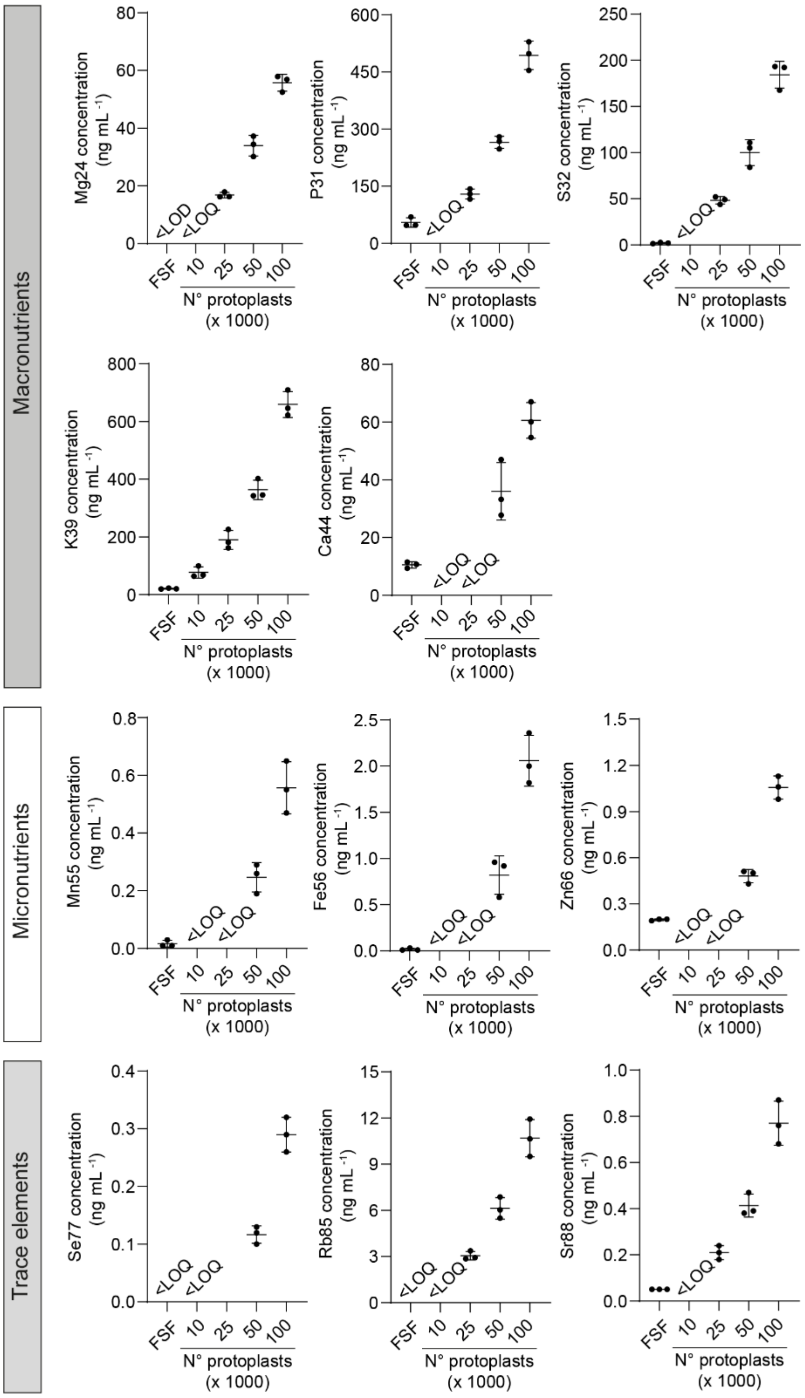

Supplementary Figure 5. Estimation of number of protoplasts required for accurate analysis of different elements. Levels of macronutrients, micronutrients and trace elements in FACS sheath fluid (FSF) without protoplasts and in sorted samples containing, 10,000, 25,000, 50,000 or 100,000 protoplasts. Sorting was performed with a BD Influx™ (BD Biosciences) sorter using 5 mM NaCl as FACS sheath fluid and element analysis with a sector field high-resolution (HR)-ICP-MS (ELEMENT 2™, Thermo Scientific™). Data are means ± SD (*n* = 3 independent samples). < LOD = indicates that the concentration was below the limit of detection of the instrument; < LOQ = indicates that the concentration could not be determined with reasonable accuracy (below limit of quantification). Source data are provided as a Source Data file.

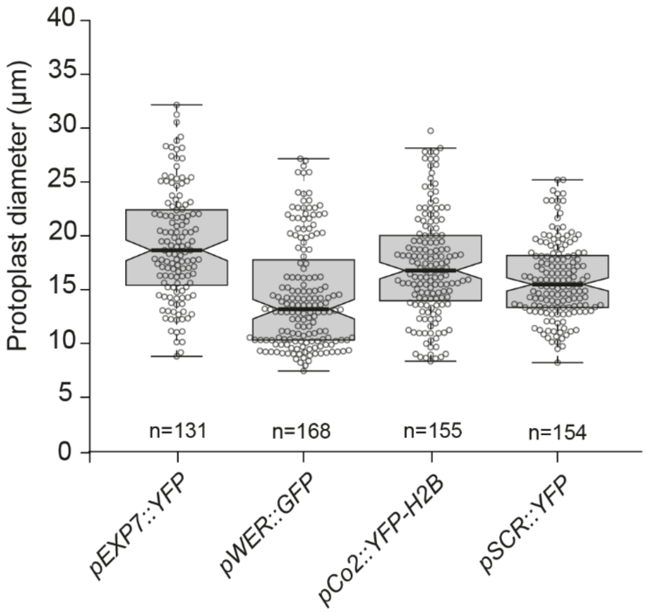

Supplementary Figure 6. Diameters of fluorescent protein-expressing protoplasts isolated from the indicated reporter lines. In the boxplots, boxes extend from the first to the third quartile around the median, while the ends of the whiskers indicate the maximum and minimum values within 1.5 x the interquartile range from the box ends. The number (*n*) of independent protoplasts analyzed is indicated. Source data are provided as a Source Data file.

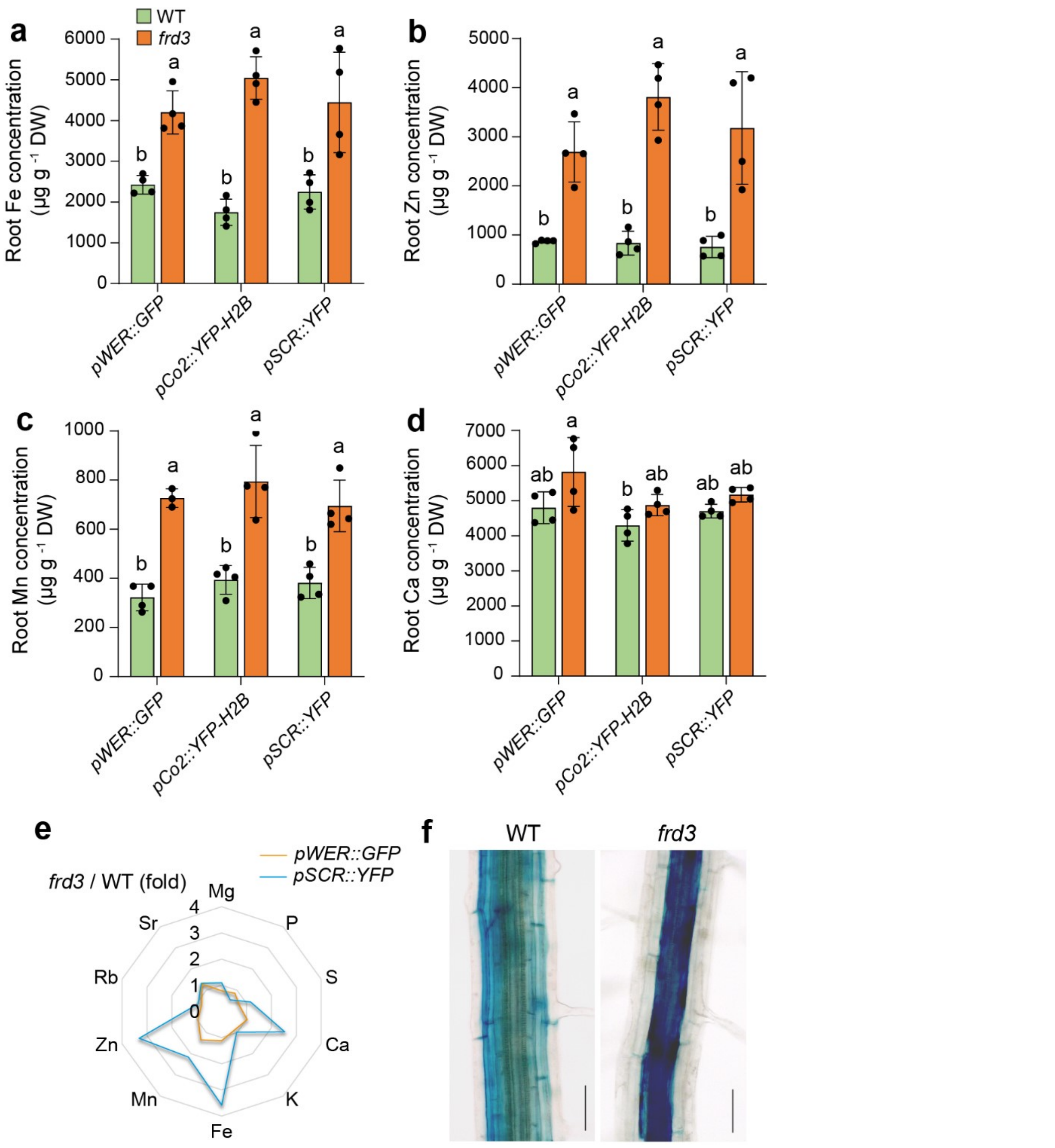

Supplementary Figure 7. Disruption of FRD3-mediated citrate loading into the xylem results in endodermal accumulation of specific elements. a-d Concentrations of Fe (**a**), Zn (**b**), Mn (**c**) and Ca (**d**) in whole roots of the indicated reporter lines in wild-type (WT) background or after introgression into *frd3* mutant background. Elemental analysis was performed on selected F3 lines. Data are means ± SD. (*n* = 4 biological replicates, except for root Mn concentration in *pWER::GFP*-expressing *frd3*, where *n* = 3 biological replicates). Different letters indicate significant differences according to one-way ANOVA followed by Tukey's test at $P < 0.05$. DW, dry weight. **e, f** Radar plots showing multi-element fold changes in sorted epidermal (*pWER::GFP*) and endodermal cells (*pSCR::YFP*) isolated from *frd3* roots relative to WT (**e**) and visualization of Fe accumulation in roots of WT and *frd3* plants with Fe-sensitive Perls staining (**f**). Seedlings were grown for 5 days on solid half-strength MS agar media. In **f**, scale bars = 50 µm. Images are representative of experiments repeated at least two times. Source data are provided as a Source Data file.

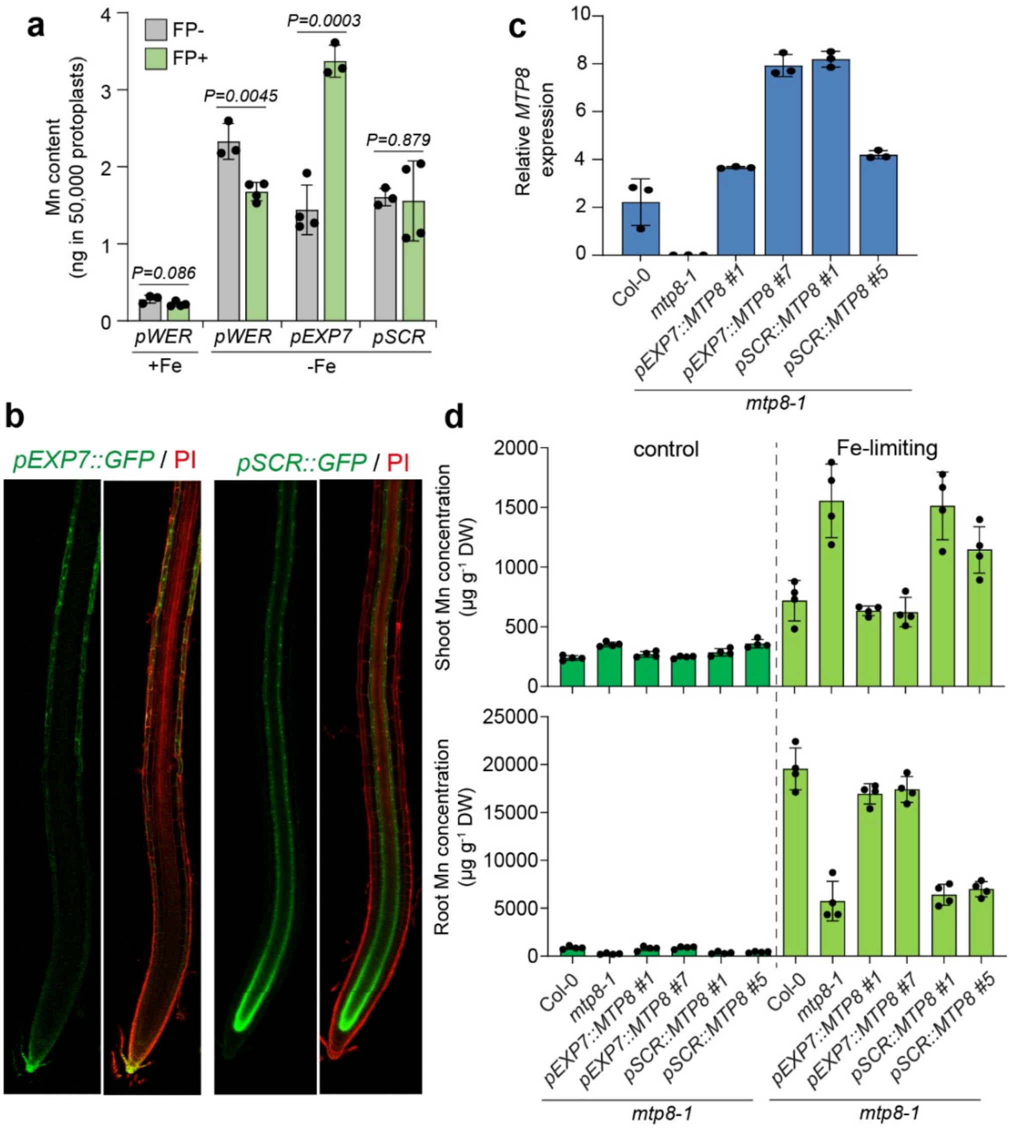

Supplementary Figure 8. Mn accumulation in *pEXP7*-expressing trichoblasts and root Mn sequestration by cell type-specific *MTP8* expression. a Mn contents in sorted fluorescence protein-positive (FP+) or negative (FP-) protoplasts. *pEXP7*, *pWER* and *pSCR* are active in trichoblasts, epidermis and endodermis, respectively. Bars show means ± SD (n = 4 independently sorted protoplasts pools, except for FP- protoplasts of *pWER* and *pSCR*, where n = 3 independently sorted protoplasts pools). P-values were determined by two-tailed unpaired Student's t test. **b** Expression domain of the promoters cloned to drive cell type-specific *MTP8* expression. Shown are images from two representative lines grown on Fe-limiting conditions. The experiment was repeated two times with similar results. **c** *MTP8* expression in roots of wild type (Col-0), *mtp8-1* and lines expressing *MTP8* under the control of *pEXP7* or *pSCR*. Plants were grown in solid half-strength MS agar media containing 30 µM Fe-EDTA and 80 µM Mn at pH 6.7 (Fe-limiting condition). Two representative lines for each construct are shown. Bars show means ± SD (n = 3 independent biological replicates). Transcript levels were calculated relative to *UBQ2*. **d** Root and shoot Mn concentrations of the indicated lines grown under control or Fe-limiting conditions. Ten-day-old seedlings were transferred to solid half-strength MS agar media containing 30 µM Fe-EDTA and 80 µM Mn at pH 5.5 (control) or pH 6.7 (Fe-limiting) and grown for 8 days. Bars show means ± SD (n = 4 independent biological replicates containing 5 plants each). DW, dry weight. Source data are provided as a Source Data file.

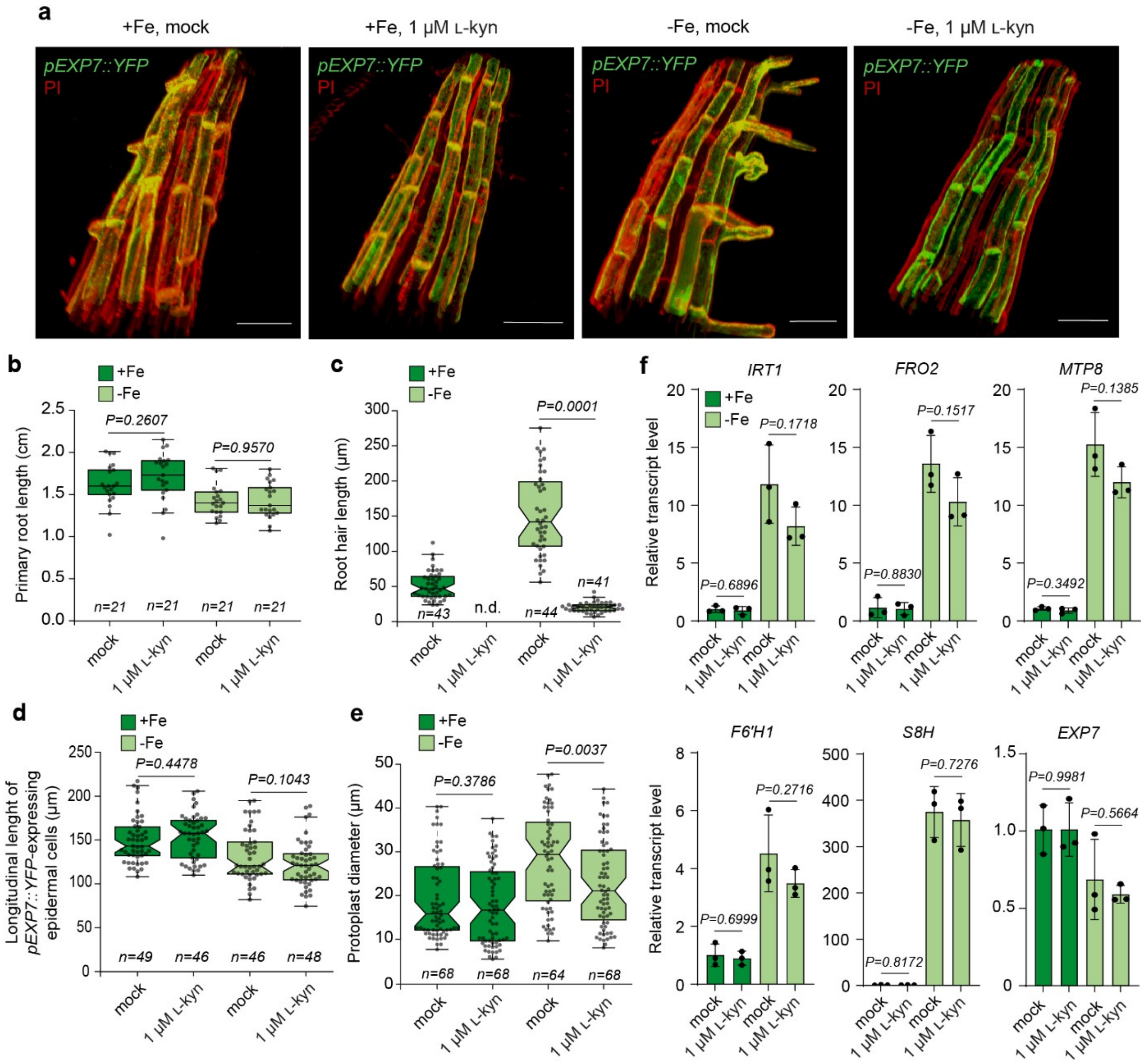

Supplementary Figure 9. Effect of L-kynurenine on root and trichoblast elongation and the expression of Fe-responsive genes. a Reconstructed confocal Z-stacks from representative roots of the *pEXP7::YFP* line grown on Fe-containing (+Fe) or Fe-depleted (-Fe) media supplemented with 1 µM L-kyn or only with the solvent DMSO (mock). Note that the *pEXP7::YFP* expression pattern in trichoblasts is not altered by L-Kyn but the elongation of hairs is prevented. Scale bars = 50 µm. **b–e** Effect of 1 µM L-kyn on the elongation of the primary root (**b**), the length of formed root hairs (**c**), the longitudinal length of *pEXP7::YFP*-expressing epidermal cells (**d**), and the size of sorted *pEXP7::YFP*-positive protoplasts (**e**). The number (*n*) of independent biological replicates analyzed is indicated. n.d., no root hairs detected. In the boxplots, boxes extend from the first to the third quartile around the median, while the ends of the whiskers indicate the maximum and minimum values within 1.5 x the interquartile range from the box ends. **f** Expression of *EXP7* and the indicated Fe deficiency-induced genes in response to L-Kyn. Seedlings were grown for 5 days on solid half-strength MS agar media containing 100 µM Fe (+Fe) or without added Fe (-Fe) and were supplemented with 1 µM L-kyn or only with the solvent DMSO (mock). Bars show means ± SD (*n* = 3 independent biological replicates). Transcript levels were calculated relative to *ACT2*. In **b–f**, *P*-values were determined by two-tailed unpaired Student's *t* test. Source data are provided as a Source Data file.

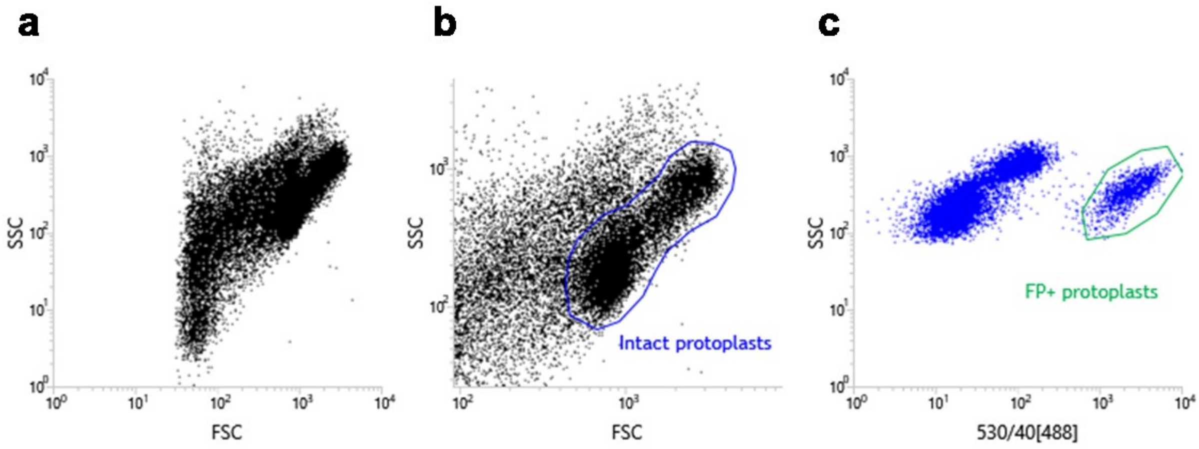

Supplementary Figure 10. Gating strategy used for fluorescence-activated cell sorting of root protoplasts. Exemplified gating strategy applied to sort protoplasts isolated from the *pSCR::YFP* reporter line. **a, b** Intact protoplasts were identified and gated in a forward scatter (FSC) vs. side scatter (SSC) dotplot using FSC as the threshold parameter (**a**, before; **b**, applied gating). **c** The fluorescent protein-positive protoplasts (FP+) were identified and gated using a 530/40 nm fluorescence vs. SSC dotplot displaying only the intact protoplasts.

Supplemental Table 1. Element concentration in solutions used for protoplast resuspension (5% mannitol), isolation (mod. solution + enzymes) and sorting (FACS sheath fluid as 5 mM NaCl). Values are expressed in ng mL^{-1}, and represent means ± s.d. (n = 4 independent replicates).

	Rb85	Sr88	Mg24	P31	S32	Ca44	Mn55	Fe56	Zn66	Li7	Cu63	K39	Se77
5% mannitol	n.d.	n.d.	4.93±0.17	7.52±0.01	4.76±0.92	23.9±1.9	0.13±0.01	0.44±0.06	n.d.	n.d.	n.d.	7.56±4.47	n.d.
Mod. solution + enzymes	27.1±0.76	19.3±0.6	4876±306	17094±1160	24305±1370	18718±1558	116.3±7.50	37.6±3.05	39.4±2.48	n.d.	42.4±3.30	26271±2944	0.46±0.11
FACS sheath fluid	n.d.	0.015±0.01	3.45±0.52	206.7±9.39	9.82±0.46	17.02±1.46	n.d.	n.d.	6.91±0.32	n.d.	2.65±0.15	50.5±5.01	n.d.

Supplemental Table 2. Element concentration in roots and shoots of cell-type reporters used in the study. Values are expressed as µg g^{-1} dry weight and represent mean ± SD (n = 4 replicates containing 20 roots or 5 shoots each). Different letters indicate significant differences among means according to Tukey's test at P < 0.05. ns, not significant according to one-way ANOVA (P < 0.05)

Root

	Ca44	Mg24	S34	P31	K39	Mn55	Fe56	Zn66	Se77	Li7	Sr88	Rb85
pWER::GFP	3708 ± 249 ns	1911 ± 108 ns	7122 ± 485 ns	7101 ± 333 ns	64726 ± 2864 b	98 ± 5.5 ns	1280 ± 147 a	521 ± 52 a	18.2 ± 0.4 b	97 ± 6.1 ns	536 ± 31.4 ns	3369 ± 200 b
pEXP7::YFP	3688 ± 48	1977 ± 73	7282 ± 255	7362 ± 197	70620 ± 3676 a	103 ± 10.4	1014 ± 19 b	512 ± 27 a	20.0 ± 0.9 a	94 ± 3.4	524 ± 15.8	3514 ± 153 a
pCo2::YFP-H2B	3491 ± 55	1859 ± 60	7084 ± 86	7251 ± 136	64620 ± 1353 b	91 ± 2.6	1219 ± 90 a	505 ± 18 ab	17.9 ± 0.3 b	91 ± 4.6	511 ± 12.3	3410 ± 110 b
pSCR::YFP	3656 ± 52	1968 ± 35	7486 ± 503	6875 ± 346	67920 ± 2328 ab	103 ± 9.0	884 ± 65 b	404 ± 79 b	18.3 ± 0.6 b	96 ± 0.6	523 ± 18.6	3189 ± 129 b

Shoot

	Ca44	Mg24	S34	P31	K39	Mn55	Fe56	Zn66	Se77	Li7	Sr88	Rb85
pWER::GFP	5214 ± 232 ns	2126 ± 115 b	7963 ± 605 ns	7047 ± 434 ns	57015 ± 2544 ns	165 ± 8.6 a	138 ± 14.1 ns	80.1 ± 5,1 b	21.4 ± 1.5 ns	279 ± 16.8 ns	699 ± 44.3 ns	2361 ± 41.6 ns
pEXP7::YFP	5194 ± 280	2061 ± 91 b	7370 ± 288	6955 ± 269	55612 ± 1990	158 ± 3.5 ab	161 ± 27.6	76.9 ± 2,5 b	20.9 ± 0.8	270 ± 20.0	686 ± 21.7	2340 ± 52.0
pCo2::YFP-H2B	5303 ± 495	2408 ± 162 a	8164 ± 334	6698 ± 329	53985 ± 2587	155 ± 13.1 ab	126 ± 5.0	79.2 ± 6,0 b	23.0 ± 1.0	271 ± 23.6	704 ± 55.3	2302 ± 29.2
pSCR::YFP	5144 ± 47	2140 ± 55 b	7814 ± 319	6895 ± 214	54864 ± 1632	142 ± 5.5 b	155 ± 16.2	98.4 ± 7,7 a	21.3 ± 0.9	259 ± 6.9	669 ± 10.0	2295 ± 26.3

Supplemental Table 3. Settings used for the analysis of different isotopes with sector field high-resolution ICP-MS.

Resolution	Isotope	Accurate mass	Method mass offset	Mass window	Mass range	Settling time	Sample time	Samples per peak	Segment duration	Search window	Integration window	Scan type	Detection mode	Integration type	IS index	IS name	Regression type	Acqu points	Peak shift
Low	Rb85	84.9113	-0.0214	120	84.741 - 85.081	0.300	0.0200	20	0.480	80	60	EScan	both	Average	3	Rh103	Linear	10	1.0
Low	Sr88	87.9051	-0.0213	120	87.729 - 88.081	0.001	0.0200	20	0.480	80	60	EScan	both	Average	3	Rh103	Linear	10	1.0
Low	Rh103	102.9050	-0.0036	120	102.699 - 103.111	0.014	0.0100	20	0.240	80	60	EScan	both	Average	-	-	Linear	10	1.0
Medium	Li7	7.0155	-0.0005	125	7.014 - 7.017	0.300	0.0100	20	0.250	60	60	EScan	both	Average	10	Rh103	Linear	10	1.0
Medium	Mg24	23.9845	-0.0019	125	23.981 - 23.988	0.071	0.0100	20	0.250	60	60	EScan	both	Average	10	Rh103	Linear	10	1.0
Medium	P31	30.9732	-0.0028	125	30.968 - 30.978	0.037	0.0100	20	0.250	60	60	EScan	both	Average	10	Rh103	Linear	10	1.0
Medium	S32	31.9715	-0.0030	125	31.967 - 31.977	0.001	0.0100	20	0.250	60	60	EScan	both	Average	10	Rh103	Linear	10	1.0
Medium	Ca44	43.9549	-0.0039	125	43.948 - 43.962	0.043	0.0300	20	0.750	60	60	EScan	both	Average	10	Rh103	Linear	10	1.0
Medium	Mn55	54.9375	-0.0044	125	54.925 - 54.946	0.038	0.0500	20	1.250	60	60	EScan	both	Average	10	Rh103	Linear	10	1.0
Medium	Fe56	55.9344	-0.0048	125	55.926 - 55.943	0.001	0.0500	20	1.250	60	60	EScan	both	Average	10	Rh103	Linear	10	1.0
Medium	Zn66	65.9255	-0.0063	125	65.915 - 65.936	0.036	0.0500	20	1.250	60	60	EScan	both	Average	10	Rh103	Linear	10	1.0
Medium	Rh103	102.9050	-0.0074	125	102.889 - 102.921	0.055	0.0500	20	1.250	60	60	EScan	both	Average	-	-	Linear	10	1.0
High	K39	38.9632	0.0005	125	38.961 - 38.966	0.300	0.1000	20	2.500	60	60	EScan	both	Average	2	Ge72	Linear	10	1.0
High	Ge72	71.9215	0.0020	125	71.917 - 71.926	0.061	0.1000	20	2.500	50	60	EScan	both	Average	-	-	Linear	10	1.0
High	Se77	76.9194	0.0010	125	76.915 - 76.924	0.001	0.3000	20	7.500	60	60	EScan	both	Average	2	Ge72	Linear	10	1.0

Supplementary Table 4. List of primers used in this study.

Cell type-specific *mtp8-1* complementation		
Fragment	Gene name	Primer sequence
promoter	*EXPANSIN7* (AT1G12560)	For: AACAGGTCTCAACCTctgtagttagatgattacaaaggggaaa<br>Rev: AACAGGTCTCATGTTtctagcctctttttctttattcttagg
promoter	*SCARECROW* (AT3G5422)	For: AACAGGTCTCAACCTtagatattgtttaagtgcatttgtga<br>Rev: AACAGGTCTCATGTTggagattgaagggttgttgg
coding sequence	*MTP8* (AT3G58060)	For: AACAGGTCTCAGGCTaaATGGAAGTCAATTATTGTCCGGAAACACC<br>Rev: AACAGGTCTCACTGATAAATCGTTGGGGATTGTAGAAAGG
real-time quantitative PCR		
UBQ2 (AT2G36170)		For: AGACGAACGCAAAGATGCAG<br>Rev: CCGGCGAAGATCAACCTCTG
ACT2 (AT3G18780)		For: GACCAGCTCTTCCATCGAGAA<br>Rev: CAAACGAGGGCTGGAACAAG
MTP8 (AT3G58060)		For: GGACAATCAGCTCCTCCAGA<br>Rev: GAAGGTATATGCACGGACGG
IRT1 (AT4G19690)		For: CGGTTGGACTTCTAAATGC<br>Rev: CGATAATCGACATTCCACCG
FRO2 (AT1G01580)		For: GCGACTTGTAGTGCGGCTATG<br>Rev: CGTTGCACGAGCGATTCTG
F6'H1 (AT3G13610)		For: TGATATCTGCAGGAATGAAACG<br>Rev: GGGTAGTAGTTAAGGTTGACTC
S8H (AT3G12900)		For: GGCACCAAATCCCTCCCAGA<br>Rev: TTTTGCCGTCGTGTGGTTGG
Genotyping by PCR		
Salk-LBa1		TGGTTCACGTAGTGGGCCATCG
HMA4Rnested		GAGATTTGGTTTTACTGCTCTGAGC
HMA4Int2F		GCAGCAGTTGTGTTCCTATTCACC
HMA4Seq1R		TGAGAGTGTGTCAAGATAATCAGC
