# Supplementary material for: Cell type-specific mapping of ion distribution in Arabidopsis thaliana roots
Source: Nat Commun. 2023 Jun 13;14:3351. doi: 10.1038/s41467-023-38880-0 (PMC10264424; doi:10.1038/s41467-023-38880-0)
Supplement: Supplementary file 3 — Reporting Summary [file 41467_2023_38880_MOESM3_ESM.pdf]

nature portfolio

Corresponding author(s): Ricardo F. H. Giehl and Nicolaus von Wirén

Last updated by author(s): May 8, 2023

Reporting Summary

Nature Portfolio wishes to improve the reproducibility of the work that we publish. This form provides structure for consistency and transparency in reporting. For further information on Nature Portfolio policies, see our Editorial Policies and the Editorial Policy Checklist.

Statistics

For all statistical analyses, confirm that the following items are present in the figure legend, table legend, main text, or Methods section.

n/a	Confirmed	
☐	☒	The exact sample size (*n*) for each experimental group/condition, given as a discrete number and unit of measurement
☐	☒	A statement on whether measurements were taken from distinct samples or whether the same sample was measured repeatedly
☐	☒	The statistical test(s) used AND whether they are one- or two-sided<br>*Only common tests should be described solely by name; describe more complex techniques in the Methods section.*
☒	☐	A description of all covariates tested
☒	☐	A description of any assumptions or corrections, such as tests of normality and adjustment for multiple comparisons
☐	☒	A full description of the statistical parameters including central tendency (e.g. means) or other basic estimates (e.g. regression coefficient) AND variation (e.g. standard deviation) or associated estimates of uncertainty (e.g. confidence intervals)
☒	☐	For null hypothesis testing, the test statistic (e.g. F, t, r) with confidence intervals, effect sizes, degrees of freedom and P value noted<br>*Give P values as exact values whenever suitable.*
☒	☐	For Bayesian analysis, information on the choice of priors and Markov chain Monte Carlo settings
☒	☐	For hierarchical and complex designs, identification of the appropriate level for tests and full reporting of outcomes
☒	☐	Estimates of effect sizes (e.g. Cohen's *d*, Pearson's *r*), indicating how they were calculated

Our web collection on statistics for biologists contains articles on many of the points above.

Software and code

Policy information about availability of computer code

Data collection	A SteREO Discovery fluorescent stereomicroscope (V8, Zeiss, Germany, an Axio Imager 2 (Carl-Zeiss) light microscope, a Axiophot 2 fluorescence microscope (Zeiss), and LSM 780 (Carl-Zeiss) or Leica SP8 (Leica Microsystems GmbH) confocal laser-scanning microscopes were used to take microscopic images. Element 2 (Thermo Fisher Scientific) HR-ICP-MS or Agilent 7900 Q ICP-MS were used for elemental analysis, as indicated in the text and in the Methods. Real-time qPCR was performed with CFX384 Touch Real-Time PCR Detection System (Bio-Rad Laboratories). Fluorescence-activated cell sorting was performed with FACSAria IIu (BD Biosciences) or a BD Influx™ (BD Biosciences) cell sorters, as detailed in the Methods.
Data analysis	All data analyses were carried out with SigmaPlot 11.0 and GraphPad Prism 9.3.1 software or the agricolae v.1.3-3 package in R. Plots were prepared with GraphPad Prism software v.9.3.1 (https://www.graphpad.com/).

For manuscripts utilizing custom algorithms or software that are central to the research but not yet described in published literature, software must be made available to editors and reviewers. We strongly encourage code deposition in a community repository (e.g. GitHub). See the Nature Portfolio guidelines for submitting code & software for further information.

Data

Policy information about availability of data

All manuscripts must include a data availability statement. This statement should provide the following information, where applicable:

- Accession codes, unique identifiers, or web links for publicly available datasets
- A description of any restrictions on data availability
- For clinical datasets or third party data, please ensure that the statement adheres to our policy

All data generated during this study are included in this published article (and its Supplementary Information File). Source data are provided with this paper.

Human research participants

Policy information about studies involving human research participants and Sex and Gender in Research.

Reporting on sex and gender	*Use the terms sex (biological attribute) and gender (shaped by social and cultural circumstances) carefully in order to avoid confusing both terms. Indicate if findings apply to only one sex or gender; describe whether sex and gender were considered in study design whether sex and/or gender was determined based on self-reporting or assigned and methods used. Provide in the source data disaggregated sex and gender data where this information has been collected, and consent has been obtained for sharing of individual-level data; provide overall numbers in this Reporting Summary. Please state if this information has not been collected. Report sex- and gender-based analyses where performed, justify reasons for lack of sex- and gender-based analysis.*
Population characteristics	*Describe the covariate-relevant population characteristics of the human research participants (e.g. age, genotypic information, past and current diagnosis and treatment categories). If you filled out the behavioural & social sciences study design questions and have nothing to add here, write "See above."*
Recruitment	*Describe how participants were recruited. Outline any potential self-selection bias or other biases that may be present and how these are likely to impact results.*
Ethics oversight	*Identify the organization(s) that approved the study protocol.*

Note that full information on the approval of the study protocol must also be provided in the manuscript.

Field-specific reporting

Please select the one below that is the best fit for your research. If you are not sure, read the appropriate sections before making your selection.

☒ Life sciences ☐ Behavioural & social sciences ☐ Ecological, evolutionary & environmental sciences

For a reference copy of the document with all sections, see nature.com/documents/nr-reporting-summary-flat.pdf

Life sciences study design

All studies must disclose on these points even when the disclosure is negative.

Sample size	We chose the sample size based on available literature in the field and our own experience in previous studies. The sample sizes used for all experiments provided sufficient resolving power to show statistical significance due to homogeneity of plant individuals.
Data exclusions	Samples were excluded from data analysis when strong deviation in plant growth after transferring from pre-culture to treatments could be clearly associated from unintentional damage caused during seedling transfer. Samples determined as outliers according to Grubbs' test (alpha = 0.05) were removed prior to data analysis.
Replication	The number of replication are indicated in the figure legends or directly on the plots. All experiments were conducted at least twice.
Randomization	All plants were grown in the same growth medium and in the same growth cabinet. The position of agar plates containing plants in the growth cabinets followed a completely randomized design.
Blinding	All the experiments were performed without prior knowledge of the final outcome, and therefore blinding was not applied.

Reporting for specific materials, systems and methods

We require information from authors about some types of materials, experimental systems and methods used in many studies. Here, indicate whether each material, system or method listed is relevant to your study. If you are not sure if a list item applies to your research, read the appropriate section before selecting a response.

Materials & experimental systems

n/a	Involved in the study
☒	☐ Antibodies
☒	☐ Eukaryotic cell lines
☒	☐ Palaeontology and archaeology
☒	☐ Animals and other organisms
☒	☐ Clinical data
☒	☐ Dual use research of concern

Methods

n/a	Involved in the study
☒	☐ ChIP-seq
☐	☒ Flow cytometry
☒	☐ MRI-based neuroimaging

Flow Cytometry

Plots

Confirm that:

☒ The axis labels state the marker and fluorochrome used (e.g. CD4-FITC).

☒ The axis scales are clearly visible. Include numbers along axes only for bottom left plot of group (a 'group' is an analysis of identical markers).

☒ All plots are contour plots with outliers or pseudocolor plots.

☒ A numerical value for number of cells or percentage (with statistics) is provided.

Methodology

Sample preparation	To isolate root protoplasts, the standard protoplasting solution 60 was replaced by a modified solution without KCl, MgCl2, CaCl2 and bovine serum albumin. The modified solution contained only 5% mannitol (w/v) buffered to pH 5.6 with 5 mM MES. This solution was used to dissolve 10 mg mL-1 cellulase "Onozuka" RS from Trichoderma viride (16420, Serva®) and 1 mg mL-1 macerozyme R-10 from Rhizopus sp. (28302, Serva®). Protoplast isolation was also efficient by using the modified solution containing only 7% mannitol (w/v) and 15-20 mg mL-1 cellulase from Trichoderma viride (Sigma Aldrich) and 3 mg mL-1 pectinase from Rhizopus sp. (Sigma Aldrich). Roots of five-day-old plants were sliced off with a razor blade. Roots were then rinsed three times with MQ water, cut into small pieces with a razor blade, and then transferred to freshly prepared protoplasting solution. Samples were incubated at 22°C for 90 min on a bench-top orbital shaker set at 80 r.p.m. After 90 min incubation in darkness, the solution containing the tissue and enzymes was aspirated with a 5mL-pipette and passed through a 40-µm cell strainer. Then, a clean pipette was used to gently aspirate the cell suspension and dispense it into cold 15-mL-screw-top tubes to a maximum of 10 mL per tube. Cell suspension was centrifuged at 500g for 5 min at 4°C to collect the protoplasts. After centrifugation, the supernatant was carefully removed without disturbing the protoplast pellet. Protoplasts were then gently resuspended in ice-cold 5% mannitol (no enzymes, no MES buffer), starting with a wide-bore (a cut-off 1-mL tip) pipette tip and then using a smaller bore (uncut 1-mL tip) pipette tip. The suspension was then transferred to a new 15-mL-screw-top tube and centrifuged at 500g for 1 min at 4°C. After removing the supernatant as described above, the procedure was repeated one more time so that the pellet was washed two times with 5% mannitol. In the final step, protoplasts were resuspended in 0.5-1.0 mL ice-cold 5% mannitol and sorted within max. 30 min.
Instrument	Protoplasts were sorted using either a FACSAria IIu (BD Biosciences) or a BD Influx™ (BD Biosciences) cell sorter.
Software	BD FACSDiva ver. 8.0.2 (FACSAria); BD FACS Sortware ver. 1.2.0.142 (BD Influx)
Cell population abundance	Under the used threshold settings (FSC) the proportion of intact protoplasts was roughly 50 % of all recorded events. The amount of fluorescent-positive protoplasts varied from 8.5 % to 65 % depending on the cell type analysed (see Fig. 2).
Gating strategy	The intact protoplasts were identified in a FSC vs. SSC dotplot using FSC as the threshold parameter. Fluorescent-positive protoplasts were identified and gated using a 530/40 nm fluorescence vs SSC dotplot displaying only the intact protoplasts. The gating strategy is exemplified in Supplementary Fig. 10.

☒ Tick this box to confirm that a figure exemplifying the gating strategy is provided in the Supplementary Information.
